# Deuteration enhances the anti-tumor effects and relative anti-inflammatory effects via affecting proliferation and apoptosis

**DOI:** 10.1016/j.heliyon.2021.e06391

**Published:** 2021-03-08

**Authors:** Ao Li, Xiaojiao Wang, Danni Li, Xiaohong Li, Rou Li, Xuejuan Yang, Xiao Li

**Affiliations:** a Department of Nuclear Medicine, Shanghai Changhai Hospital, Shanghai 200433, China; b Southwest University of Science and Technology, Mianyang 621010, China; c Department of Cardiology, Yinchuan Second People's Hospital, Yinchuan 750004, China

**Keywords:** Apigenin, Deuteration, Apoptosis, Cell proliferation, Anti-inflammation

## Abstract

Apigenin (AP) is a plant flavonoid with potential biomedical applications. To enhance the anti-tumour effect, AP was deuterated via hydrogen–deuterium exchange under hydrothermal conditions. The anti-tumor effects of deuterated AP (D-AP) were then tested on HCT116 cells and on a murine model of turpentine-induced inflammation. Cell cycle progression and cell proliferation were measured by flow cytometry, and *in vivo* immuno-inflammation was evaluated by quantitating glucose metabolism using ^18^F-fluorodeoxyglucose positron emission tomography. According to the mass spectral results, the efficiency of AP deuteration was 62.96%. For both the two groups of AP and D-AP at 24 h and 48 h, there were an obvious increase on perception of G2 phage. Apigenin showed the ability of blocking in G2 phage to inhibit cellular proliferation. Additionally, D-AP induced early apoptosis in more cells than did AP (12.1% vs. 10.4%). Moreover, D-AP induced a more severe process of anti-inflammation during the early period, resulting in a more effective anti-inflammatory response. Therefore, given the innate ability of D-AP to block cell proliferation and induce early apoptosis, we conclude that deuteration enhances the systemic anti-cancer effect of this flavonoid.

## 1 Introduction

Many specific and efficient flavonoid compounds have been explored and applied in food and drug development owing to their high bioactivities [1, 2, 3]. Apigenin (AP; 4ʹ,5,7-trihydroxyflavone), a readily available plant flavonoid and the aglycone of several naturally occurring glycosides, has shown anti-tumour effects both *in vitro* and *in vivo* [4]. During tumour inhibition, AP acts in two ways [5, 6]. On the one hand, it controls tumour cell proliferation (including apoptosis or autophagy induction), cell cycle regulation, and migration or invasion inhibition. For example, it inhibits the proliferation of pancreatic cancer cells by arresting the G_2_/M phase of the cell cycle [7]. On the other hand, the flavonoid indirectly affects tumour progression by stimulating the systemic immune response, such as by inhibiting the expression of nitrix oxide synthase, cyclooxygenase, and pro-inflammatory cytokines [8, 9].

AP is highly conducive to medicinal development owing to its low toxicity and low mutagenicity. However, because of its low solubility and high permeability, it is categorised as a Biopharmaceutics Classification System Class II drug, meaning that requires a relatively longer effect period and higher uptake efficiency [1, 10, 11].

Deuteration, in which a hydrogen atom in a small molecule is replaced by deuterium, is an easy way to improve the bioavailability of biopharmaceuticals as well as to avoid extensive systemic waste in drug delivery, as the kinetic isotope effect results in a lower rate of metabolism of the deuterated drug molecule, allowing more time for it to take effect [12, 13]. As the first batch of US Food and Drug Administration-approved deuterated drugs, deuterated tetrabenazine (Austedo) has shown superior effects to its non-deuterated form in treating Huntington's chorea [14]. Unlike the change in effects resulting from a change of functional groups, hydrogen–deuterium exchange (HDX) in small-molecule drugs strengthens the drug's effectiveness by retarding its rate of metabolic breakdown [15]. Throughout the drug's distribution and re-distribution period, deuterium reduces the drug's pharmacokinetics by decreasing its metabolic efficiency and thus its frequency or dosage. During oxidation in Phase 1 metabolism, carbon–deuterium bonds are harder to break, thus further decreasing the metabolic rate of the drug and prolonging its effective period. Some encouraging clinical outcomes with deuterated anti-tumour drugs have been achieved; for example, sorafenib, ceritinib, and enzalutamide, which are currently being tested in clinical trials, have exhibited longer half-lives and lower effective doses [16, 17]. Additionally, HDX can reduce the formation of toxic metabolites, thereby increasing the biosafety of drugs [18]. Hence, HDX is a promising strategy for enhancing the anti-tumour effect of AP.

This study focused on the systemic anti-tumour effect of deuterated AP (D-AP), including its influence on tumour cell proliferation, apoptosis induction, and immuno-inflammatory responses. The objectives were to gain a comprehensive understanding on the improvement of drug effects through deuteration and to provide some reference for the clinical transformation of D-AP.

## 2 Materials and methods

### 2.1 Deuteration of apigenin

For the deuteration of AP, 0.5 g of the flavonoid was added to 15 mL of heavy water (D_2_O), and the pH of the solution was adjusted to being slightly alkaline using sodium deuteroxide (NaOD) solution. The AP solution was sealed in a hydrothermal reactor at 180 °C for 10 h, following which the product was lyophilised to extract D-AP_1_. This D-AP_1_ extract was then also deuterated by adding 0.5 g to 15 mL of D_2_O. After adjusting the pH to a slightly alkaline level using NaOD solution, the D-AP_1_ solution was sealed in a hydrothermal reactor at 200 °C for 10 h, following which the product was lyophilised to extract D-AP_2_. The D-AP_2_ extract was deuterated one more time, using the same method described above, to yield D-AP_3_.

### 2.2 Mass spectrometry

Mass spectra of D-AP_1_, D-AP_2_, and D-AP_3_ were recorded using an Agilent InfinityLab LC/MSD system to quantify the ratios of D-AP.

### 2.3 Cell counting kit-8 for HCT116

The human colon cancer cell line HCT116 was supplied by BoGoo BioTech Co., Ltd (Shanghai, China). The cells were cultured in Dulbecco's modified Eagle's medium containing 10% foetal bovine serum at 37 °C in a 5% CO_2_ atmosphere. Cells at the logarithmic phase were then digested with trypsin and re-seeded onto 96-well plates. After 24 h, the medium was replaced with medium containing AP at different concentration. The cells were divided into the following treatment groups with AP concentrations of 5, 10, 20, 40, 80, 160 and 320 μmol/L and one control group with fresh medium.

### 2.4 Influence of deuteration on the tumour cell-inhibiting activity of apigenin

Cells at the logarithmic phase were then digested with trypsin and re-seeded onto 6-mm discs. Once the cells had adhered to the plate wall, they were divided into the following five treatment groups: the control group with no extra component added; the AP group that was incubated with 80 μmol/L AP for 24 h; the AP group that was incubated with 80 μmol/L AP for 48 h; the D-AP group that was incubated with 80 μmol/L D-AP_3_ for 24 h; the D-AP group that was incubated with 80 μmol/L D-AP_3_ for 24 h.

The numbers of cells in the various cell cycle phases (i.e., G_1_/G_0_, S, or G_2_/M) after the treatment period were measured by flow cytometry using a propidium iodide (PI) staining kit according to the manufacturer's instructions. The percentage of cells in each phase was quantified with the flow cytometry histogram.

Flow cytometry was also applied for the enumeration of cells in apoptosis, using an Annexin V-fluorescein (FITC)/PI staining kit according to the manufacturer's instructions. A logarithmic scatter plot based on Annexin V-FITC (X) versus PI (Y) fluorescent signals was obtained as a four-quadrant diagram, where each quadrant represented the percentage of the total number of cells examined, with the upper left quadrant (K1) being for cell loss occurring in the process of collecting cells, the upper right quadrant (K2) being for late apoptotic cells and necrotic cells, the lower left quadrant (K3) being for living cells, and the lower right quadrant (K4) being for early apoptotic cells.

### 2.5 Inflammation model set-up

All animal experiments in this study were approved by the Institutional Animal Care and Use Committee of Shanghai Changhai Hospital and carried out in accordance with the institutional guidelines of Shanghai Changhai Hospital. A murine model of inflammation was set up using female Kunming mice (6 weeks old; *c.a.* 20 g in weight). The mice were reared on a normal diet under specific-pathogen-free conditions. After the mice had been acclimatised to the environment, 70 mL of turpentine oil was injected into the medial muscle of the left posterior thigh to induce an inflammatory abscess. Immediately after the turpentine injection, a 20 mg/kg dose of AP or D-AP_3_ was administered to the mice in the respective groups via tail vein injection [19]. For the control group, 0.01 M phosphate-buffered saline was injected instead. When the mice were of difficulty in walking and the tissue of treated leg was of swelling, the inflammation model was successfully set up.

### 2.6 Evaluation of the in vivo anti-inflammatory effect

On the one hand, the size of the lesion was measured to understand the effects of AP on acute inflammation. Specifically, the left and right hind thigh peripheries of each mouse were recorded before and at 24 and 72 h after drug or saline administration to assess the swelling reduction rates. On the other hand, to evaluate glucose metabolism during the early anti-inflammatory response, mice were subjected to ^18^F-fluorodeoxyglucose positron emission tomography (^18^F-FDG PET) after 24 h of drug or saline administration. The PET scan was performed 30 min after the injection of ^18^F-FDG at 3.7 MBq/20 g. The maximum standardised uptake value (SUV_max_) was recorded to determine the degree of glucose metabolism by the lesion.

### 2.7 Statistical analysis

All experiments were repeated three times and the results are expressed as the mean ± standard deviation. Paired t-tests were performed between AP and corresponding D-AP groups, and differences with *p* < 0.05 were considered to be statistically significant.

## 3 Results

### 3.1 Deuteration of apigenin

**Figure 1:**
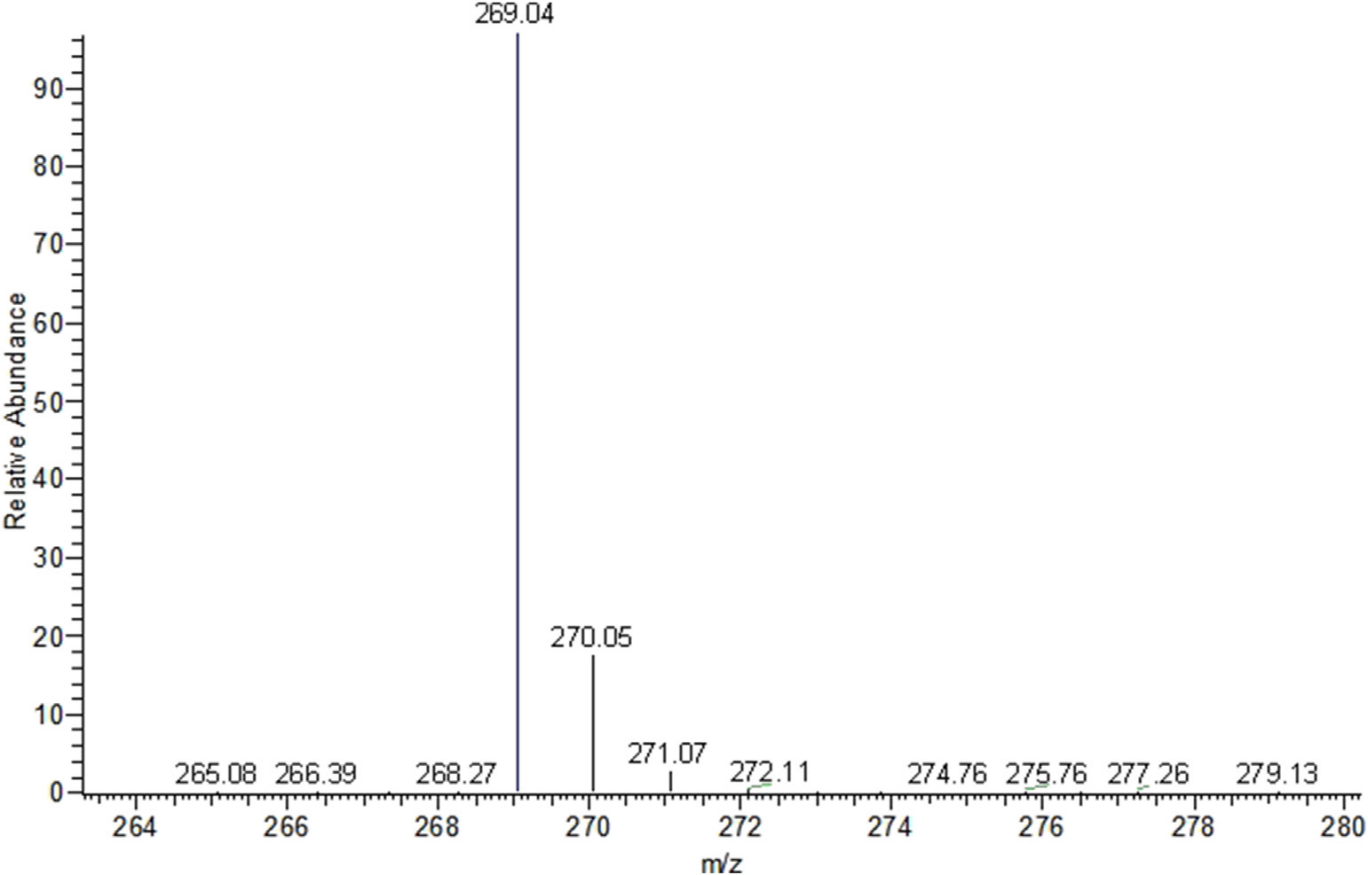
The spectrum of apigenin that was measured with ESI nagative method.

**Figure 2:**
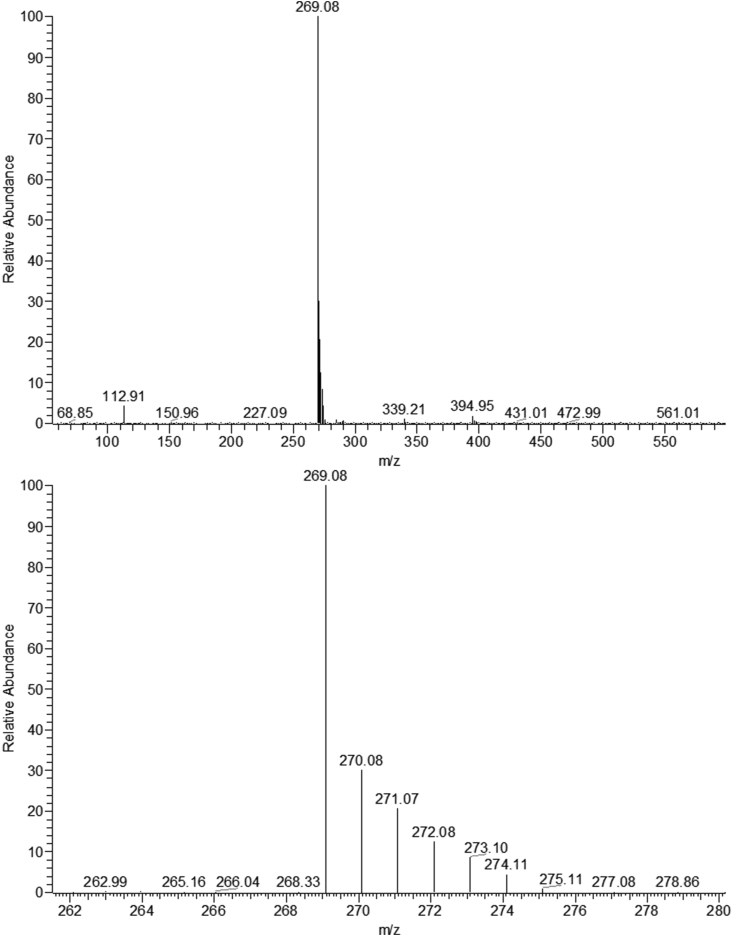
The spectrum of Deuterated apigenin and the spectrum of D-AP specified around M/Z = 270.

The AP molecule has 10 hydrogen atoms that could be replaced by deuterium. Under hydrothermal conditions, 51.69%, 58.85%, and 62.96% of the hydrogen atoms of AP were deuterated after the first (D-AP_1_), second (D-AP_2_), and third (D-AP_3_) HDX reactions, respectively. The MS spectrum of apigenin and deuterated apigenin that used in the following experiments were provided in Figure 1 and Figure 2. Because of the chemical inertness of some of the hydrogen atoms, the ratio of deuteration was approximately 60%, meaning that the D-AP solution used in the study was a mixture of AP and D-AP.

### 3.2 Influence of deuteration on HCT116 cell proliferation

**Figure 3:**
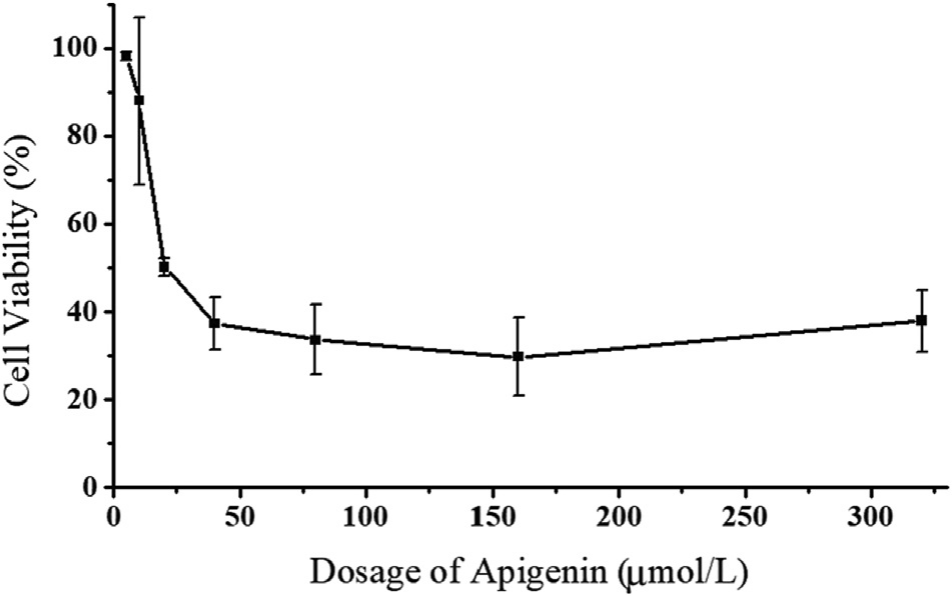
The cell viability of HCT116 cells after 24 h AP treatment.

The line diagram in Figure 3 showed the results of CCK8 cytotoxicity test on HCT116 cells. As shown in Figure 3, cell activity decreased along with the increase of AP dose. In the low-dose treatment groups (≦40 μmol/L), cell activity decreased rapidly and slowed down after the concentration reaching 80 μmol/L, the cell activity of 80 μmol/L group was 33.61 ± 8%. Subsequently, with the increase of treatment dose, the tendency of cell activity decrease did not maintain. At higher doses (>80 μmol/L), the additive effect of dosage on the therapeutic effect of AP was not significant, potentially resulted from the long period incubation.

**Figure 4:**
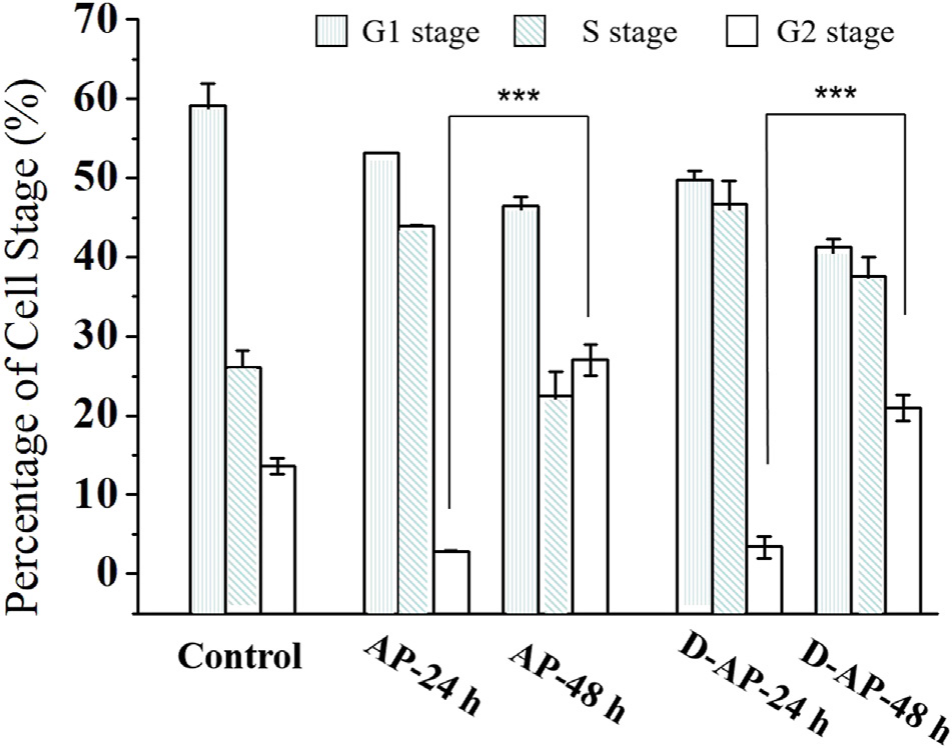
Proportion of HCT116 cells at each stage after 24 h or 48 h co-incubation with 80 μmol/L of the drugs, including the flow cytometry data of HCT116 cells without treatments, the flow cytometry data of 80 μmol/L apigenin (AP)- or deuterated apigenin (D-AP)-treated cells with 24 h treatments, and the flow cytometry data of 80 μmol/L apigenin (AP)- or deuterated apigenin (D-AP)-treated cells with 48 h treatments.

The diagram in Figure 4 showed the proportion of cell phages of the control group, AP group and D-AP group for 24 h and 48 h. The proportion of AP group at 24 h in S phase was significantly higher than the control group cells (43.98 ± 0.07% vs 26.11 ± 2.06%, *p* < 0.05). The proportion of AP group at 48 h in G2 phase was significantly higher than the AP group at 24 h (26.99 ± 1.97% vs 2.88 ± 0.04%, *p* < 0.05). The results manifested that AP had affection for the cell cycle of proliferation, where the cell cycle was blocked at S phase and G2 phase. Based on the above results, AP was inferred of a further affect on G2 phase than on S plate in the cell cycle.

By comparing the data of the proportion of the AP group at 24 h and 48 h, D-AP group at 24 h and 48 h, the proportion of D-AP group at 24 h in S phase was significantly higher than the AP group cells (46.76 ± 2.80% vs 43.98 ± 0.07%, *p* < 0.05). Meanwhile, the proportion of D-AP group at 48 h in G1 phase and G2 phase were significantly lower than the AP group at 48 h in G1 (41.51 ± 1.02% vs 46.42 ± 2.80%, *p* < 0.05) and G2 phase (20.96 ± 1.70% vs 26.99 ± 1.97%, *p* < 0.05).

**Figure 5:**
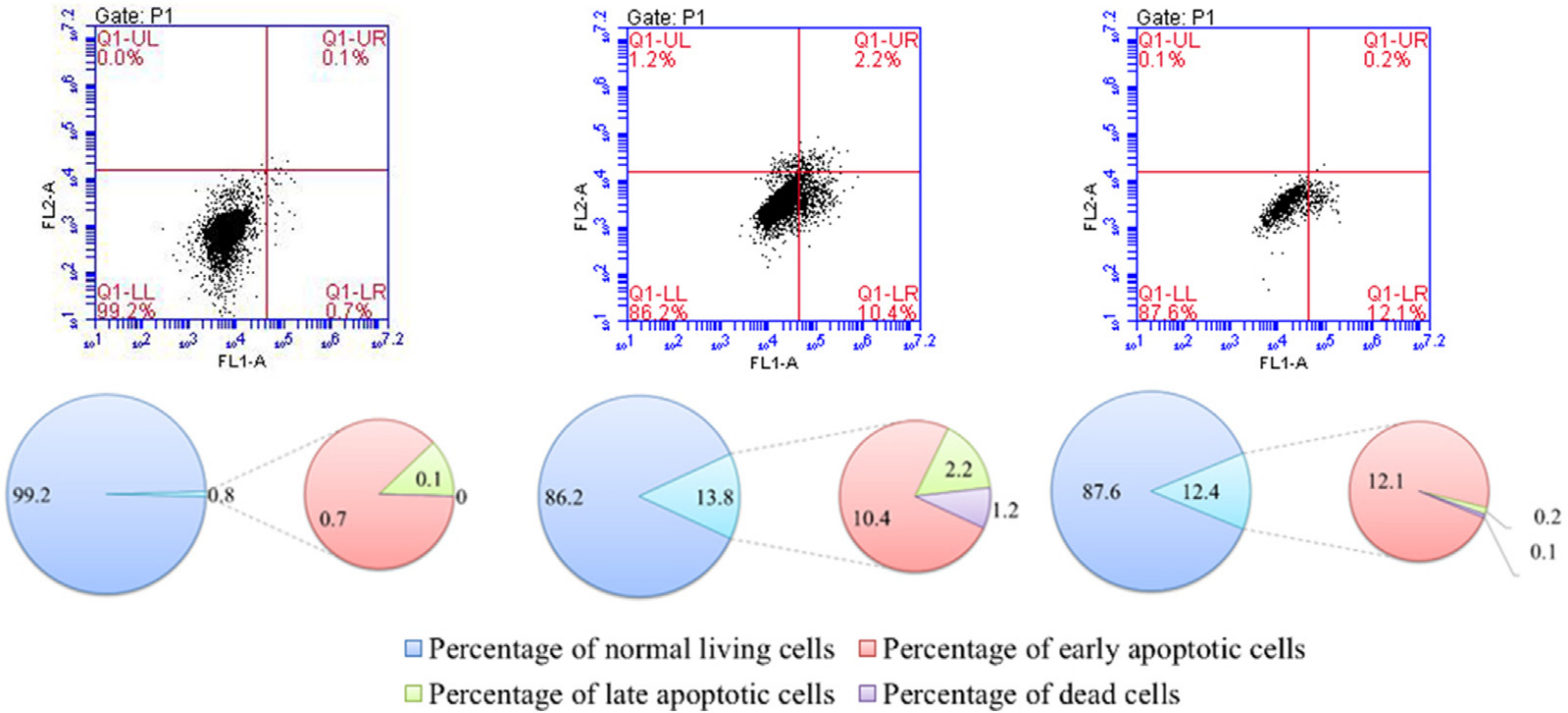
Cellular apoptosis after 24 h co-incubation with 1× PBS (A), 80 μmol/L apigenin (AP) (B), or 80 μmol/L deuterated apigenin (D-AP) (C). AP and D-AP induced apoptosis of the HCT116 cells, where a higher proportion of early apoptosis was induced by D-AP (12.1%) than by AP (10.4%).

AP also affects tumour cells by inducing the apoptotic process, and HCT116 cells are more likely to undergo apoptosis in the presence of this flavonoid. After 24 h co-incubation with 80 μmol/L of either AP or D-AP, the rate of apoptosis and hence the number of dead HCT116 cells had increased to more than 12% (Figure 5). The rate of early apoptosis was 10.4% for the AP-treated cells and 12.1% for the D-AP-treated cells, but the difference was not statistically significant. However, the rate of late apoptosis induced by D-AP was lower than that by AP.

### 3.3 Influence of deuteration on immuno-inflammation

**Figure 6:**
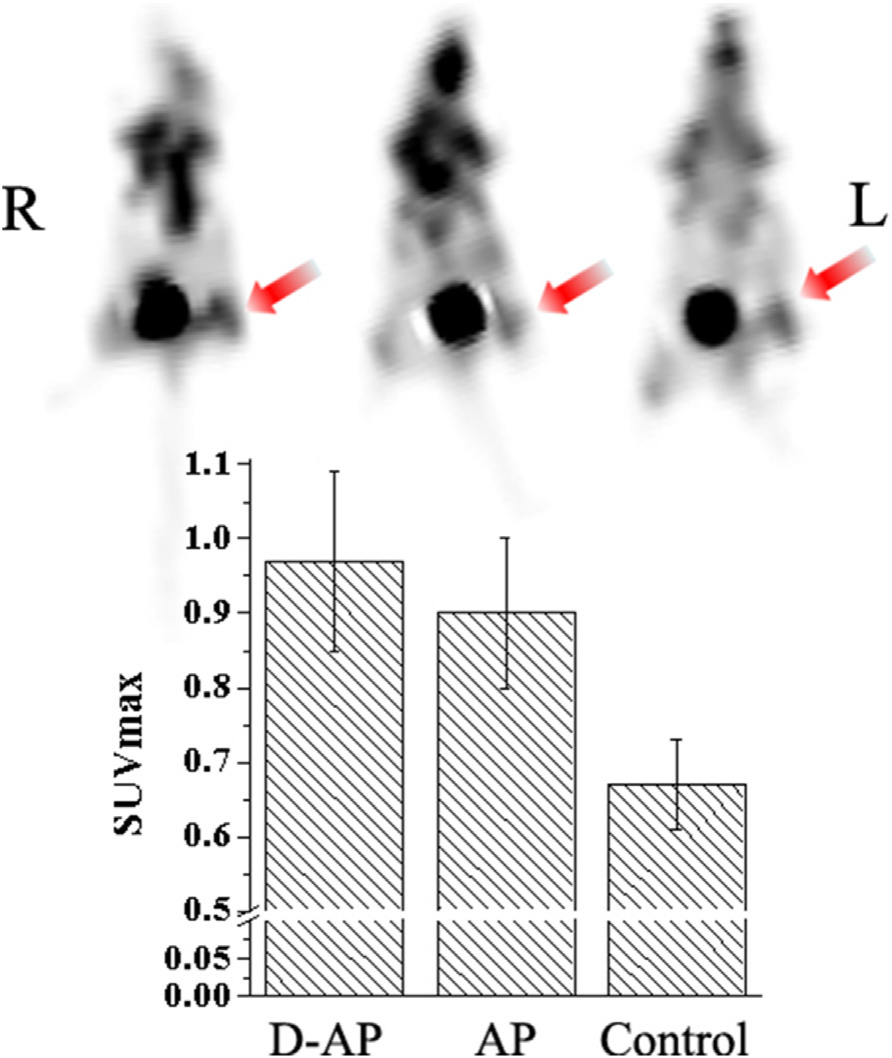
^18^F-FDG PET scans showing the intensity of immuno-inflammation reactions at 24 h (early period). In deuterated apigenin (D-AP)-treated mice, lesions consumed more glucose owing to the more severe anti-inflammatory response induced. R and L represent the right and left sides of the mouse, respectively.

**Figure 7:**
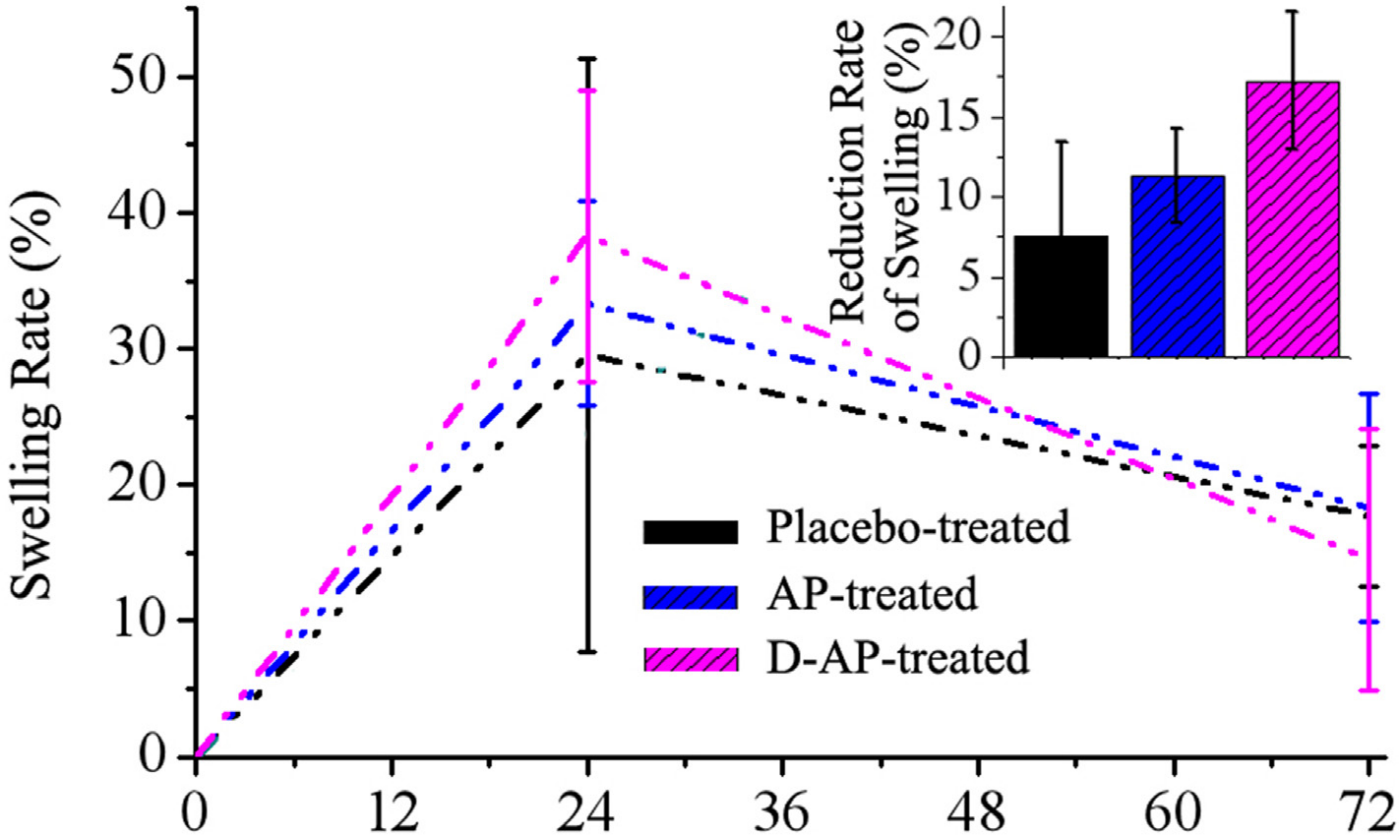
Changes in the perimeter of the affected limb after the treatments. There was a higher rate of swelling reduction between 24 h (early period) and 72 h (later period).

Besides its anti-tumour effect, AP also has an effect on immuno-inflammation events *in vivo*. When AP was used to treat turpentine-induced inflammation in mice, an interesting phenomenon was observed. Based on the ^18^F-FDG PET scans at 24 h post intravenous injection of the drugs (Figure 6), the D-AP group had the highest local SUV_max_, indicating a more severe anti-inflammatory process that expended more energy from glucose metabolism. The results from the longer (72 h) observation of perimeter changes in the affected limbs (Figure 7) corresponded with the ^18^F-FDG PET results. Owing to the more severe anti-inflammatory response induced by D-AP, the mice in this group had the highest swelling rate in the early period (within 24 h) post injection. More importantly, however, the D-AP-treated mice showed a higher rate of swelling reduction from 24 to 72 h (17.2 ± 4.3%). During the whole process, difficulty in walking was not observed for all these groups.

In fact, AP had affected the degree of turpentine-induced inflammation in the mice throughout the period of establishment of the inflammation model. After 24 h of drug administration (the early period), the degree of inflammation induced by D-AP had increased most. After 72 h of administration (later period), the D-AP-treated mice showed the highest rate of swelling reduction and more thorough recovery from the turpentine-induced inflammation, indicating that the deuteration of AP had significantly enhanced its anti-inflammatory effect, albeit the differences in the therapeutic effects of the three groups were not statistically significant.

## 4 Discussion

The triple HDX reactions under high-temperature and high-pressure conditions in the hydrothermal reactor resulted in a relatively high ratio of D-AP (62.96%). Compared with other methods, the hydrothermal method has higher practicability and repeatability.

AP inhibited the proliferation of cancer cells by causing arrest in the G_1_ phase of the cell cycle. In tumour cells, AP has been found to reduce the expression of cyclinD1, a key factor in cancer cell formation [20]. In HCT116 cells, apigenin treatment could induce cell to arrest at G2/M phase to inhibit cell growth and associated with an increase of cell cycle inhibitors, p53 and p21WAF1/CIP1 [21]. These growth-inhibiting effects were further enhanced the deuteration. In addition, AP down-regulates the expression of Bcl-2 in apoptotic cells and up-regulates that of the Bax protein, thereby inducing apoptosis of the HCT116 cells. Although AP and D-AP induced apoptosis at similar rates, AP induced early apoptosis at a significantly lower rate (*p* < 0.05). Based on this difference in early apoptosis, D-AP may induce apoptosis for a longer time.

Inflammation is an autoimmune process in biological systems. The degree of this process is heightened in the presence of AP. We verified that AP exacerbated the process of inflammation in mice in the early stages of treatment and increased both the scale of inflammation in the inflammatory model and the swelling rate of the left hind leg. Therefore, glucose metabolism at 24 h was higher than that under normal conditions owing to the increased energy consumption by the lesion, indicating that the immune process was more intense and deuteration of the drug had promoted this process further.

In conclusion, deuteration enhances the anti-tumour effect of AP, which means that a lower effective dosage of the drug can be used with less biological toxicity and side-effects.

## Declarations

### Author contribution statement

Ao Li: Conceived and designed the experiments; Performed the experiments; Contributed reagents, materials, analysis tools or data; Wrote the paper.

Xiaojiao Wang: Conceived and designed the experiments.

Danni Li; Xiaohong Li: Performed the experiments.

Rou Li; Xuejuan Yang: Analyzed and interpreted the data.

Xiao Li: Contributed reagents, materials, analysis tools or data; Wrote the paper.

### Funding statement

This work was supported by the 10.13039/501100001809National Natural Science Foundation of China (Grant No. 81701761).

### Data availability statement

Data will be made available on request.

### Declaration of interests statement

The authors declare no conflict of interest.

### Additional information

No additional information is available for this paper.
